# Antioxidant Performance and Characterization Comparison of Carbon Dots Derived from Agricultural Waste Pineapple Peel

**DOI:** 10.3390/foods15020189

**Published:** 2026-01-06

**Authors:** Zhaoqi Pan, Yiyang Zhou, Binghong Ji, Qining Liu, Ziluan Fan

**Affiliations:** 1 State Key Laboratory of Woody Oil Resources Utilization, Northeast Forestry University, Harbin 150040, China; 2023222236@nefu.edu.cn (Z.P.); 2023222254@nefu.edu.cn (Y.Z.); jbh_0901@nefu.edu.cn (B.J.); 2023222123@nefu.edu.cn (Q.L.); 2 College of Life Science, Northeast Forestry University, Harbin 150040, China

**Keywords:** carbon quantum dots, dopants, pineapple peel waste, microwave method, structural characterization, antioxidant, antibacterial activity

## Abstract

This study prepared carbon dots (CDs) from agricultural waste pineapple peel via an eco-friendly microwave method, optimizing their performance through copper ion and chitosan doping. Multiple characterization techniques and performance tests were employed for systematic analysis. Antioxidant assays revealed that PP-CDs have excellent concentration-dependent free radical scavenging activity: the DPPH IC_50_ values of Pineapple Peel Carbon Dots (PP-CDs), Copper-Doped Pineapple Peel Carbon Dots (Cu-PP-CDs) and Chitosan-Doped Pineapple Peel Carbon Dots (CS-PP-CDs) are 0.79, 0.95 and 0.98 mg/mL, while their ABTS IC_50_ values are 0.22, 0.40 and 0.26 mg/mL, respectively. Antibacterial tests showed modified CDs have enhanced activity: Cu-PP-CDs exhibit inhibition zones of 23.1 ± 0.13 mm (*E. coli*) and 17.3 ± 0.05 mm (*S. aureus*) with MICs of 2.5 and 5.0 mg/mL, while CS-PP-CDs have respective zones of 12.8 ± 0.08 mm and 16.3 ± 0.12 mm with a 5.0 mg/mL MIC for both strains. All CDs present a quasi-spherical morphology and emit yellow fluorescence under UV excitation, with PP-CDs showing the strongest intensity. This study provides technical support for high-value utilization of pineapple peel and development of multifunctional CDs, which have food field potential but face large-scale production and toxicological evaluation challenges.

## 1. Introduction

With the global push for carbon peaking and carbon neutrality, the consensus on green, low-carbon, and circular agricultural development, and the growing demand for agricultural waste recycling, the green and cost-effective production of novel functional nanomaterials has emerged as a key research focus in materials science and environmental studies. CDs, a cutting-edge carbon nanomaterial, typically measure less than 10 nm in size. This material not only has a simple synthesis method and stable chemical properties, but also has good hydrophilicity, good biocompatibility, low toxicity and excellent specific surface area [1]. With these advantages, it is outstanding among many nanomaterials and is widely used in nanobiomedicine, functional materials, photoelectric, catalytic chemistry and biosensing [2,3,4]. However, traditional carbon dot preparation methods often rely on expensive chemical raw materials or complex processes, which not only have high costs but also may have negative impacts on the environment. Therefore, the development of green, low-cost and efficient carbon dot synthesis methods has become an important direction of current research [5].

Agricultural waste has gradually become an ideal carbon source for the preparation of CDs due to its wide source, strong renewable and rich carbon content [6]. By transforming agricultural waste into high value-added CDs, it can not only realize the recycling of resources, but also reduce environmental pollution, which is in line with the concept of green chemistry and sustainable development [7,8]. As a major by-product of pineapple processing, pineapple peel is rich in cellulose, polysaccharides and natural antioxidants, which provides a unique advantage for its use as a raw material for carbon dot preparation [9]. In addition, the efficient use of pineapple peel can provide a new way to solve the problem of agricultural waste accumulation [10].

This study utilizes pineapple peel as a carbon source to prepare CDs through a simple and efficient microwave method. By doping with metal ions or natural polymers, the electronic structure and surface functional groups of CDs can be further regulated to enhance their functional properties. Copper doping has been proven to significantly improve the antibacterial activity of CDs, while the introduction of chitosan may enhance their biocompatibility and targeting efficiency [11,12]. In this study, we innovatively constructed three distinct systems, namely pristine PP-CDs, Cu-PP-CDs, and CS-PP-CDs. By systematically comparing their microstructures, surface functional groups, and core functional properties including fluorescence performance, antioxidant activity and antibacterial efficacy, we clarified the regulatory effects of different doping modification approaches on the properties of PP-CDs. The microwave method has the advantages of short reaction time, low energy consumption and simple operation. It is a green and environmentally friendly synthesis method [13] to develop a low-cost, high-performance multifunctional carbon dot material by systematically characterizing its physicochemical properties and evaluating its fluorescence performance, antioxidant activity, and antibacterial effect [14].

## 2. Materials and Methods

### 2.1. Materials, Reagents and Equipment

Fresh pineapple peels sourced from Xuwen County, Guangdong Province, were purchased from Biyoute Supermarket and stored at ambient temperature; the materials and reagents used included food-grade 95% ethanol supplied by Tianjin Tianli Chemical Reagents Co., Ltd. (Tianjin, China), biological-grade (98%) DPPH free radicals and ABTS free radicals from Beijing Boao Tuoda Technology Co., Ltd. (Beijing, China), chitosan provided by Shanghai Aladdin Biochemical Technology Co., Ltd. (Shanghai, China), as well as analytical-grade (99.0%) sodium dihydrogen phosphate, sodium bicarbonate, ascorbic acid, sodium chloride, and copper sulfate all obtained from Tianjin Tianli Chemical Reagents Co., Ltd. (Tianjin, China), alongside deionized water; the experimental equipment comprised an FW100 type crusher from Tianjin Tester Instrument Co., Ltd. (Tianjin, China), a PC20MSW type microwave oven manufactured by Guangdong Midea Kitchen Appliances Manufacturing Co., Ltd. (Foshan, China), an SHB-III S type circulating water multi-purpose vacuum pump from Zhengzhou Great Wall Science & Technology Trading Co., Ltd. (Zhengzhou, China), an RE-2000A type rotary evaporator and a YRE-2000A type water bath both from Gongyi Yuhua Instrument Co., Ltd. (Gongyi, China), an LT-07 type dried fruit machine from Zhongshan Youxike Electric Co., Ltd. (Zhongshan, China), a DHG-9140A type forced-air drying oven supplied by Shanghai Yiheng Scientific Instrument Co., Ltd. (Shanghai, China), a 79-1 type magnetic heating stirrer from Changzhou Danrui Experimental Equipment Co., Ltd. (Changzhou, China), a TGL-16C type compact benchtop centrifuge manufactured by Changzhou Maikeno Instrument Co., Ltd. (Changzhou, China), a JA2003N type electronic analytical balance from Shanghai Youke Instrument Co., Ltd. (Shanghai, China), an Epoch type full-wavelength microplate reader from BioTek USA (Budapest, Hungary), a Nicolet iN10 type Fourier transform infrared spectrometer from Thermo Fisher Scientific USA (Carlsbad, CA, USA), a JEM-2100F type transmission electron microscope and a SmartLab SE type X-ray diffractometer both from Rigaku Corporation (Tokyo, Japan), and an X-ray photoelectron spectrometer from Thermo Fisher Nexus (Singapore). 

### 2.2. Carbon Dot Synthesis Process

#### 2.2.1. Preparation of PP-CDs

Refer to the method of Chaiwarit et al. [15] prepare PP-CDs, fresh pineapple peels were rinsed thoroughly with deionized water for three consecutive times to eliminate surface contaminants. The cleaned peels were then placed in a fruit dryer for 36 h of drying, and finally ground to a particle size of 80 mesh.

Weighed 10 g of crushed pineapple peel and mixed it thoroughly with 50 mL of deionized water. The mixture was transferred to a beaker, which was then placed in a microwave oven operating in pure microwave mode at a power of 800 W and processed for 8 min. The beaker was taken out for thorough stirring every 2 min during the process, until all moisture evaporated, yielding a brown solid product.

The beaker was placed in a dark place and cooled to room temperature. Then, 70 mL of deionized water was added to dissolve the solid material, followed by the addition of ethanol solution at a volume three times that of the aqueous solution for alcohol precipitation to remove uncarbonized macromolecular impurities. The mixture was transferred to a rotary evaporator and concentrated at 60 °C. Subsequently, the concentrated mixture was centrifuged at 4000 r/min for 10 min. After vacuum filtration through a 0.22 μm filter membrane, 10 mL of the carbon dot solution was transferred into a dialysis bag with a molecular weight cutoff of 1000 Da. The dialysis bag was immersed in a beaker containing 2 L of deionized water, and the beaker was placed on a magnetic stirrer for continuous stirring during dialysis, which lasted for a total of 24 h. The dialysis solution was rapidly replaced every 6 h, with a total of 4 replacements. Upon completion of dialysis, the resulting dialysis solution was collected and transferred to a 100 mm Petri dish, which was then placed in an oven and dried at 40 °C for 24 h. After drying, the solid product formed in the Petri dish was scraped off to obtain carbon dot solid powder, which was stored in a refrigerator at 4 °C for subsequent use.

#### 2.2.2. Preparation of Cu-PP-CDs

Following the methodology reported by Liu et al. [16], the pineapple peel carbon dot (PP-CDs) solution was prepared in strict accordance with their experimental protocol. A 1 mol/L copper sulfate solution was prepared using a 50 mL volumetric flask: 7.98 g of copper sulfate was accurately weighed, dissolved in a suitable volume of deionized water, and then the resulting solution was transferred to the 50 mL volumetric flask; deionized water was added to bring the solution up to the calibration mark, followed by thorough mixing to obtain a homogeneous solution for subsequent use. From this prepared stock solution, 2 mL was aliquoted and added to the as-prepared carbon dot solution. The mixture was thoroughly homogenized on a magnetic stirrer, then heated in a water bath at 70 °C for 10 min. After cooling to room temperature, the mixture was subjected to centrifugation followed by vacuum filtration. The treated mixture was then transferred into a dialysis bag for dialysis treatment, with the deionized water refreshed every 6 h and the dialysis process maintained for a total of 24 h. The resulting copper-doped pineapple peel carbon dot (Cu-PP-CDs) solution was finally transferred to an oven for drying. Extensive experiments have demonstrated that copper-doped carbon dots exhibit extremely low toxicity with negligible biological hazards [17,18]. As documented in the relevant literature [19], after copper species are doped and anchored onto the surface of CDs, they form stable coordination complexes with the hydroxyl, carboxyl, and other functional groups on the carbon dot surface. This effectively reduces the concentration of free copper ions, thereby significantly mitigating the biological toxicity of the material.

#### 2.2.3. Preparation of CS-PP-CDs

The PP-CDs solution was first prepared following the established experimental protocol. Subsequently, 0.3 g of chitosan was accurately weighed and dissolved in 50 mL of 1% *v*/*v* acetic acid aqueous solution, with thorough agitation to yield a homogeneous chitosan solution. The purified PP-CDs solution was mixed with the as-prepared chitosan solution at a 1:1 volume ratio, and the resultant mixture was subjected to microwave irradiation at the identical power setting for 2 min to ensure the complete integration of chitosan and CDs. After cooling to ambient temperature, the mixture was processed via centrifugation and filtration using the procedures described previously. The treated mixture was then transferred into a dialysis bag for dialysis treatment; the deionized water used for dialysis was refreshed every 6 h, and the dialysis process was maintained for 24 h to eliminate residual acetic acid from the system. Finally, the resulting chitosan-doped pineapple peel carbon dot solution was transferred to an oven for drying to constant weight.

### 2.3. Antioxidant Activity Assay

#### 2.3.1. DPPH Free Radical Scavenging Test

Adopting the methodology of Joga et al. [20], weigh a specified amount of DPPH and prepare a stock solution at 0.25 mg/mL (approximately 0.1 mM). Dilute the stock solution to achieve an absorbance of 0.70 ± 0.02 at 517 nm before use to ensure stable solution activity. Dissolve dried PP-CDs powder in deionized water to prepare a stock solution at 2.0 mg/mL. Perform gradient dilution to prepare CDs solutions at 0.2 mg/mL, 0.4 mg/mL, 0.6 mg/mL, 0.8 mg/mL, 1.0 mg/mL, 1.2 mg/mL, 1.4 mg/mL, 1.6 mg/mL, 1.8 mg/mL and 2.0 mg/mL. Calculate the DPPH radical scavenging rate using Equation (1).

(1) $$DPPH\;Free\;Radical\;Scavenging\;Rate\;(\%)=[1-\frac{A_{1}-A_{2}}{A_{0}}]\times 100\%$$

In the formula, the absorbance of the carbon dot-DPPH mixture at 517 nm is denoted as A_1_; the absorbance of the DPPH stock solution at 517 nm is denoted as A_0_; and the absorbance of the carbon dot–water mixture at 517 nm is denoted as A_2_.

Note that the DPPH stock solution was prepared with ethanol as the solvent, and all absorbance determinations were conducted after confirming that the mixed system was uniformly dispersed without obvious phase separation or turbidity. The blank correction strategy was carried out with A_2_ to eliminate the background absorbance interference derived from carbon dot solutions themselves. The linear absorption range of the prepared DPPH solution at 517 nm was verified in advance to ensure that all absorbance values involved in calculation fell within the valid linear range, thus ensuring the accuracy and reliability of the test results.

The DPPH radical scavenging experiments for Cu-PP-CDs and CS-PP-CDs composites were conducted as described above.

#### 2.3.2. ABTS Free Radical Scavenging Test

Adopting the methodology of Wang et al. [21], mix 7 mmol ABTS solution with 2.45 mmol potassium persulfate solution in a 1:1 ratio, then store the mixture in a dark place at room temperature for 16 h to prepare ABTS^+^ radical stock solution. Dilute with PBS buffer (pH 7.4) to achieve an absorbance of 0.70 ± 0.02 at 734 nm before use. Dissolve dried PP-CDs powder in deionized water to prepare a 0.5 mg/mL stock solution. Perform gradient dilution to obtain 0.05 mg/mL, 0.10 mg/mL, 0.15 mg/mL, 0.20 mg/mL, 0.25 mg/mL, 0.30 mg/mL, 0.35 mg/mL, 0.40 mg/mL, 0.45 mg/mL mg/mL and 0.5 mg/mL carbon dot solutions. Calculate the ABTS radical scavenging rate using Equation (2):

(2) $$ABTS\;Cation\;Radical\;Scavenging\;Rate\;(\%)=[1-\frac{A_{1}-A_{2}}{A_{0}}]\times 100\%$$

In the formula, the mixture of ABTS and PBS buffer is designated as A_0_; the mixture of carbon dot solution and ABTS is designated as A_1_; and the mixture of CDs with PBS buffer is designated as A_2_.

The experiments on Cu-PP-CDs and CS-PP-CDs for ABTS radical scavenging were conducted as described above.

### 2.4. Antibacterial Activity

#### 2.4.1. Inhibition Zone Test

In reference Anugraha et al. [22], the plate-well method was adopted to evaluate the inhibitory effects of PP-CDs, Cu-PP-CDs and CS-PP-CDs against *Escherichia coli* and *Staphylococcus aureus*. Briefly, 100 µL of the bacterial suspension was uniformly spread onto the agar medium. Wells with an approximate diameter of 6 mm were punched into the medium using a micropipette, and 60 µL of the test sample solution was added to each well. For experimental controls, cefotaxime and chitosan solutions were used as the positive controls, while deionized water served as the negative control, with equal volumes (60 µL) added to the corresponding wells. The inoculated plates were then incubated at 37 °C for 24 h, following which the diameters of the inhibition zones were measured via the cross-impregnation method.

#### 2.4.2. Minimum Inhibitory Concentration Assay

Follow the method of Yan, Z [23], bacterial strains in the logarithmic growth phase were diluted to a concentration of 1.5 × 10^8^ CFU/mL. Aliquots of 100 µL of the diluted bacterial suspension and 100 µL of three carbon dot solutions with varying concentrations were added to a 96-well microplate, resulting in a final volume of 200 µL per well. The initial well was loaded with a carbon dot solution at a concentration of 40 mg/mL, which was then subjected to a 2-fold serial dilution for 6 consecutive gradients, yielding a final concentration of 0.625 mg/mL. Deionized water was used as the negative control. The microplate was then incubated at 37 °C for 24 h in a constant-temperature incubator. The minimum inhibitory concentration (MIC) was determined according to the following protocol: after incubation, colony counting was performed for each well, and the lowest concentration of carbon dots corresponding to wells with no visible colony growth was tentatively defined as the MIC of the sample. To ensure the reliability of the results, three sets of parallel experiments were conducted. The growth status of colonies in the negative control wells was monitored simultaneously, and the experimental system was deemed valid only when robust colony growth was observed in the negative control wells. Finally, the bactericidal efficacy of different carbon dot samples was evaluated via the colony counting method.

### 2.5. Characterization of CDs

The elemental composition and content analysis was performed using an X-ray photoelectron spectroscopy (XPS) instrument (Singapore). The acquired XPS spectrum data was processed with Avantage 6.9 for peak fitting, enabling precise characterization of the elemental types and their relative abundances on the carbon dot surface [24].

The material’s microstructure and lattice characteristics were characterized by X-ray diffraction. A Cu target was used as the radiation source, with scans conducted across the 5–80 angular range. The resulting diffraction patterns were analyzed to determine whether the CDs were crystalline or amorphous.

The morphology, particle size and dispersion of CDs were observed by transmission electron microscopy.

Surface functional group analysis was performed using a Fourier transform infrared (FTIR) (Carlsbad, CA, USA) spectrometer. Infrared spectra were measured across the 400–4000 cm^−1^ wavenumber range, with characteristic absorption peaks assigned to identify surface functional groups such as hydroxyl and carboxyl groups on CDs. Fluorescence characterization was conducted using a fluorescence spectrophotometer (Waltham, MA, USA). By setting different excitation wavelengths, corresponding emission spectra were measured and recorded to analyze the excitation-dependent fluorescence properties and emission patterns of the CDs.

## 3. Results

### 3.1. Antioxidant Activity Analysis

**Figure 1:**
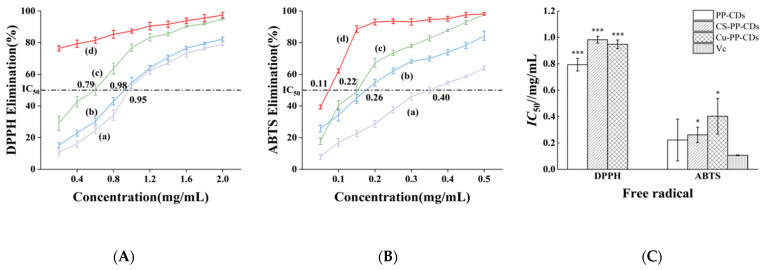
Antioxidant activity of pineapple peel-based carbon dots. (**A**): DPPH Radical Scavenging Rate; (**B**): ABTS^+^ Radical Scavenging Rate. (**C**): IC_50_ values of pineapple peel-based carbon dots and vitamin C against different free radicals. n = 3; *: *p* < 0.05; ***: *p* < 0.001. a—Cu-PP-CDs; b—CS-PP-CDs; c—PP-CDs; d—VC.

As shown in Figure 1, the antioxidant properties of three different CDs are presented.

The antioxidant activities of three types of PP-CDs including Cu-PP-CDs, CS-PP-CDs and PP-CDs as well as vitamin C (VC) were investigated, with the half-maximal inhibitory concentration IC50 as the core evaluation index. The results exhibited significant substance specificity and free radical type dependence. In terms of DPPH radical scavenging activity, as observed from the scavenging curves and IC50 histogram, the IC50 values of the three PP-CDs were all higher than that of vitamin C, with differences among them: the IC50 of Cu-PP-CDs was 0.95 mg/mL, CS-PP-CDs was 0.98 mg/mL and PP-CDs was 0.79 mg/mL. This indicated that unmodified PP-CDs had a slightly superior DPPH radical scavenging capacity compared with Cu-PP-CDs and CS-PP-CDs, while vitamin C, as a classic antioxidant, showed stronger DPPH scavenging efficacy with an IC50 much lower than that of the carbon dot materials. Meanwhile, the DPPH radical scavenging rate of the CDs increased in a dose-dependent manner with the rise in concentration; at a concentration of 2.0 mg/mL, the DPPH scavenging rate of PP-CDs approached 90%, demonstrating the potential of PP-CDs to effectively scavenge DPPH radicals. For ABTS^+^ radical scavenging activity, the IC50 values of the CDs decreased significantly: the IC50 of Cu-PP-CDs was 0.40 mg/mL, CS-PP-CDs was 0.26 mg/mL and PP-CDs was 0.22 mg/mL, which indicated that the three CDs all had a greatly enhanced ABTS^+^ radical scavenging capacity, and the scavenging efficacy of CS-PP-CDs and PP-CDs was close to that of vitamin C with an IC50 of 0.11 mg/mL.

DPPH is a lipid-soluble free radical and ABTS^+^ is a water-soluble free radical, and the hydrophilic and hydrophobic properties of CDs may determine their effect on different types of free radicals. The hydrophilic surface of PP-CDs may be more compatible with the water-soluble environment of ABTS^+^, thus exhibiting better scavenging capacity. In addition, compared with vitamin C, a traditional antioxidant, PP-CDs were slightly inferior in DPPH scavenging efficacy but showed ABTS^+^ scavenging activity close to that of vitamin C. Moreover, CDs possess good biocompatibility and degradability, which provides possibilities for their application in the fields of food antioxidant and biomedicine.

### 3.2. Antibacterial Performance Analysis

The table below shows the comparison of bacteriostatic diameters between the carbon deposition sites of pineapple peel and their control groups.

#### Antibacterial Test Results and Conclusions

For the antibacterial tests, three parallel replicates were conducted for each group.

As shown in Table 1, the antibacterial test results demonstrated that PP-CDs modified by copper ion and chitosan doping had significantly enhanced antibacterial performance, with the antibacterial zones around the doped samples being notably larger than those around the undoped pure CDs. Notably, Cu-PP-CDs exhibited the largest antibacterial zone diameter and the most remarkable antibacterial effect, and both doped CD types outperformed the undoped pure PP-CDs in all aspects of antibacterial performance.

Experiments on the inhibition of Escherichia coli showed that Cu-PP-CD samples presented the most transparent and well-defined inhibition zones with a maximum diameter of 23.1 ± 0.13 mm; the inhibition zone diameter of CS-PP-CD samples was 12.8 ± 0.08 mm, while pure PP-CDs produced no inhibition zone. These quantitative results confirmed that copper doping exerted potent bactericidal effects against Gram-negative bacteria. Using cefotaxime as the positive control, its inhibition zone diameter against *E. coli* was 34.2 ± 0.17 mm, the inhibition zone diameter of the chitosan control group was 10.2 ± 0.21 mm and the normal saline control group showed no antibacterial effect.

The antibacterial inhibition effect against *Staphylococcus aureus* showed a consistent pattern. Cu-PP-CD composites displayed the largest inhibition zone of 17.3 ± 0.05 mm, the inhibition zone diameter of CS-PP-CD composites was 16.3 ± 0.12 mm and pure PP-CDs also produced no inhibition zone. Cefotaxime had an inhibition zone diameter of 35.1 ± 0.06 mm against *Staphylococcus aureus*, the chitosan control group was 8.7 ± 0.22 mm and the normal saline control group had no antibacterial activity. Notably, all three CD types produced larger inhibition zones against *Escherichia coli* than against *Staphylococcus aureus*, indicating their superior strain-specific targeting efficacy toward *Escherichia coli* [25].

The minimum inhibitory concentration results were highly consistent with those of the inhibition zone tests. Cu-PP-CD composites showed the lowest MIC value of 2.5 mg/mL against *Escherichia coli*, indicating significant antibacterial efficacy, and their MIC value against *Staphylococcus aureus* reached 5.0 mg/mL. CS-PP-CDs maintained an MIC value of 5.0 mg/mL against both bacterial strains, outperforming pure PP-CDs in both cases.

The comprehensive results of the inhibition zone experiments and quantitative data indicated that doping modification, especially copper ion doping, significantly improved the antibacterial efficacy of PP-CDs. This modification method provides a practical path for the high-value utilization of agricultural by-products such as pineapple peels and also offers new ideas for the development of low-cost, environmentally friendly antibacterial materials. Such agricultural waste-based antibacterial materials may have the potential to be explored for applications in food preservatives [26], food packing [27], antimicrobial agents research and development in the future [28].

### 3.3. Characterization Analysis

#### 3.3.1. Carbon Dot Morphology Analysis

**Figure 2:**
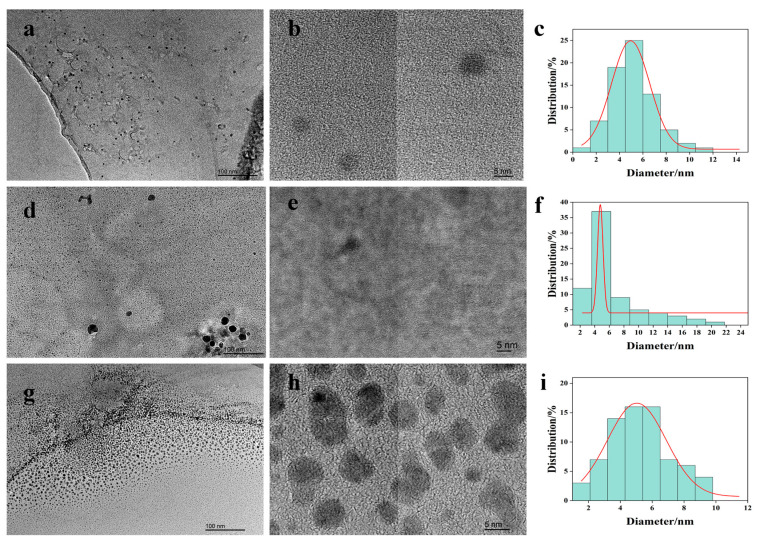
TEM image of pineapple peel-based carbon dots. PP-CDs (**a**) Low-magnification TEM image (scale 100 nm), (**b**) High-magnification TEM image (scale 5 nm), (**c**) Particle size distribution (n = 70); Cu-PP-CDs, (**d**) Low-magnification TEM image (scale 100 nm), (**e**) High-magnification TEM image (scale 5 nm), (**f**) Particle size distribution (n = 70); CS-PP-CDs, (**g**) Low-magnification TEM image (scale 100 nm), (**h**) High-magnification TEM image (scale 5 nm), (**i**) Particle size distribution (n = 70).

Shown in Figure 2 are the morphologies of three different CDs observed under a transmission electron microscope.

The low-magnification TEM image of pure PP-CDs (Figure 2a, scale bar: 100 nm) showed that the CDs existed in a dispersed state with no obvious agglomeration. In the high-magnification TEM image (Figure 2b), they exhibited a spherical nanoscale morphology with a clear lattice structure and no obvious impurity phases, indicating that the prepared PP-CDs possessed excellent monodispersity and crystallinity. The particle size distribution histogram (Figure 2c) demonstrated that the particle size of PP-CDs ranged from 2 to 12 nm, with the peak centered at approximately 4 nm and a narrow particle size distribution coefficient, confirming the good size uniformity of the pure CDs.

In the TEM images of Cu-PP-CDs (Figure 2d,e), the CDs still maintained a spherical morphology, but slight particle aggregation was observed in some areas compared with pure PP-CDs. This might be attributed to the altered interfacial forces between particles after copper ions coordinated with the surface functional groups of the CDs. The particle size distribution histogram (Figure 2f) showed that the particle size range of Cu-PP-CDs broadened to 2–24 nm, with the peak at about 4 nm, and a small number of large particle (10–24 nm) appeared. It is speculated that the doping of copper introduced a local coordination polymerization effect, leading to slight agglomeration of some carbon dot particles and thus the broadening of the particle size distribution [29].

The TEM images of CS-PP-CDs (Figure 2g,h) indicated that CS-PP-CDs retained a complete spherical morphology and exhibited better dispersibility than Cu-PP-CDs, with only a small amount of small-scale agglomeration. A thin amorphous layer was observed on the surface of the CDs in the high-magnification image, which was an organic layer formed by chitosan grafted onto the carbon dot surface via covalent bonds or hydrogen bonds, confirming the successful composite of chitosan and CDs. The particle size distribution histogram (Figure 2i) showed that the particle size of CS-PP-CDs ranged from 2 to 12 nm, with the peak centered at approximately 6 nm, which was slightly larger than that of pure PP-CDs. This was due to the grafting of chitosan molecular chains increasing the apparent particle size of the CDs, while the particle size distribution coefficient remained narrow, indicating that chitosan doping did not destroy the size uniformity of the CDs but instead improved the dispersion stability of the particles through surface modification.

In summary, all three types of PP-CDs exhibited a spherical nanomorphology; pure PP-CDs had the optimal monodispersity and size uniformity. Copper doping caused slight agglomeration due to coordination, resulting in the broadening of particle size distribution. Chitosan doping achieved a moderate increase in particle size and an improvement in dispersion stability through surface grafting. The differences in morphology and particle size directly reflected the regulatory effect of different doping methods on the microstructure of CDs: the coordination of copper ions mainly affected the aggregation behavior of CDs, while the grafting of chitosan regulated the particle size and interfacial properties of CDs through surface modification. These microstructural characteristics provide a morphological basis for subsequent interpretation of the structure–activity relationship between the antioxidant and antibacterial properties of CDs and their structures [30]. The chitosan coating also enables surface functionalization with targeting ligands, rendering them advantageous in the biomedical field as drug carriers for targeted delivery and controlled release of drugs [31].

#### 3.3.2. Comparative Analysis of Carbon Dot Lattice Structures

**Figure 3:**
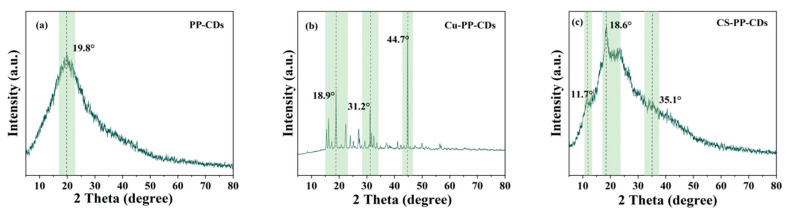
X-ray diffraction patterns of PP-CDs (**a**), Cu-PP-CDs (**b**), and CS-PP-CDs (**c**).

Shown in Figure 3 are the X-ray diffraction patterns of three different CDs.

PP-CDs exhibit a broadened diffraction peak at 2θ = 19.8°, a characteristic peak of amorphous or graphite-like structures in carbon materials [32]; it shows that it is mainly amorphous carbon, and only a local graphite-like short-range ordered structure exists, and the lattice order degree is medium.

Through standard reference pattern matching analysis, the Cu-PP-CDs composite exhibits multiple sharp crystalline diffraction peaks at 2θ = 18.9°, 31.2° and 44.7°, confirming the presence of crystalline copper-based compounds within the material. Copper ion doping effectively induces crystalline phase formation and significantly enhances the lattice orderliness of the material. Consistent with X-ray photoelectron spectroscopy experimental data, including characteristic peaks of the Cu 2p orbital and elemental composition analysis, copper ions have been successfully doped into the carbon dot system, with the aforementioned crystalline diffraction peaks further corroborating the effectiveness of the doping process.

The diffraction peaks of CDs incorporated with chitosan exhibit broader and more diffuse characteristics, showing weak broad peaks at 2θ = 11.7°, 18.6° and 35.1°. As chitosan is an amorphous or low-crystallinity polymer polysaccharide, its incorporation into carbon dot composites further disperses the ordered graphite-like structure of the CDs, resulting in a significant reduction in overall lattice order.

In summary, the three materials exhibit distinct differences in the lattice order and structure of carbon dot-based systems due to their inherent variations in doping or composite components: PP-CDs demonstrate a localized graphite-like short-range order; the ordered structure of CDs doped with chitosan is further disrupted; while copper ion doping enhances lattice order through the introduction of crystalline phases.

#### 3.3.3. Carbon Dot Optical Properties

**Figure 4:**
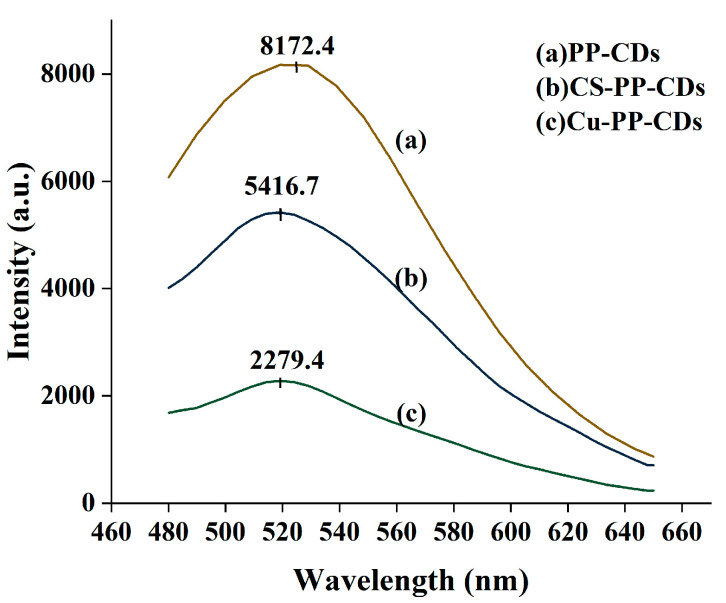
Fluorescence spectra of PP-CDs, Cu-PP-CDs and CS-PP-CDs.

Shown in Figure 4 are the fluorescence emission spectra of three distinct CDs.

**Figure 5:**
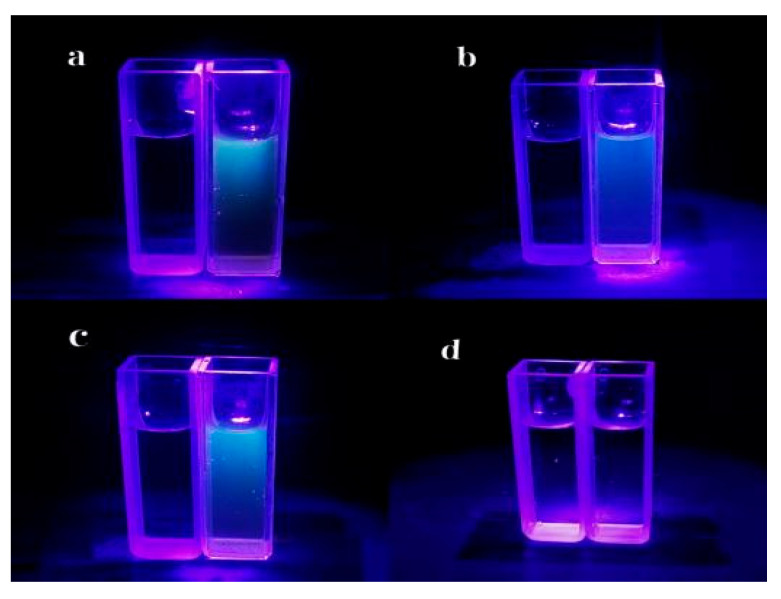
Contrast image of 365 nm ultraviolet excitation fluorescence of PP-CDs, Cu-PP-CDs and CS-PP-CDs. (**a**) Fluorescence comparison of PP-CDs and water; (**b**) Fluorescence contrast of Cu-PP-CDs and water; (**c**) Fluorescence contrast of CS-PP-CDs with water; (**d**) Water versus water fluorescence contrast.

Shown in Figure 5 are the UV-fluorescence comparison spectra of three different CDs.

From the fluorescence spectra and visual fluorescence characterization results under excitation with 365 nm ultraviolet light, it is evident that the fluorescence emission characteristics of PP-CDs, CS-PP-CDs, and Cu-PP-CDs exhibit a distinct gradient difference. Pristine PP-CDs display the highest fluorescence emission intensity of 8172.4 a.u. at approximately 520 nm; after chitosan doping, the fluorescence emission intensity of the CDs decreases to 5416.7 a.u., and the fluorescence emission intensity of Cu-PP-CDs further attenuates to 2279.4 a.u. This change in data directly reflects that doping modification can significantly regulate the fluorescence emission capacity of CDs, and the regulatory range of copper elements on the fluorescence intensity of CDs is much larger than that of chitosan, with a quantitative difference in the effects of the two on the fluorescence properties of CDs.

In the visual fluorescence comparison experiment excited by 365 nm ultraviolet light (Figure 5), different groups present fluorescence phenomena highly consistent with the spectral data. Group a is the fluorescence comparison between PP-CDs and water; the PP-CDs solution shows bright blue fluorescence under ultraviolet light, forming a strong visual contrast with the non-fluorescent state of water, which directly confirms that pristine PP-CDs possess excellent fluorescence luminescent properties. Group b is the fluorescence comparison between Cu-PP-CDs and water; the Cu-PP-CDs solution only exhibits weak blue fluorescence, and its fluorescence brightness is much lower than that of PP-CDs in group a, further verifying the significant inhibitory effect of copper doping on the fluorescence emission intensity of CDs. Group c is the fluorescence comparison between CS-PP-CDs and water; the fluorescence brightness of the CS-PP-CDs solution is between that of group a and group b, indicating that the inhibitory degree of chitosan doping on the fluorescence intensity of CDs is significantly weaker than that of copper doping. Group d is the fluorescence comparison between water and water; as a blank control group, this group shows no obvious fluorescence signal, which effectively excludes the interference of fluorescence generated by water itself and ensures the reliability and specificity of the fluorescence detection results of the three groups of carbon dot samples.

Combining the fluorescence spectral data and the visual characterization results under 365 nm ultraviolet light, it can be concluded that both chitosan and copper doping lead to a decrease in the fluorescence emission intensity of PP-CDs, with a clear differentiation in the degree of their effects. The essence of this phenomenon can be attributed to the reconstruction of the electronic conjugated structure of CDs after doping, the change in the coordination binding state of surface functional groups, and the differential regulation of the charge transfer path and efficiency inside the CDs, ultimately resulting in a gradient attenuation of the energy transfer efficiency during the fluorescence emission process.

#### 3.3.4. Comparative Analysis of Surface Functional Groups of CDs

**Figure 6:**
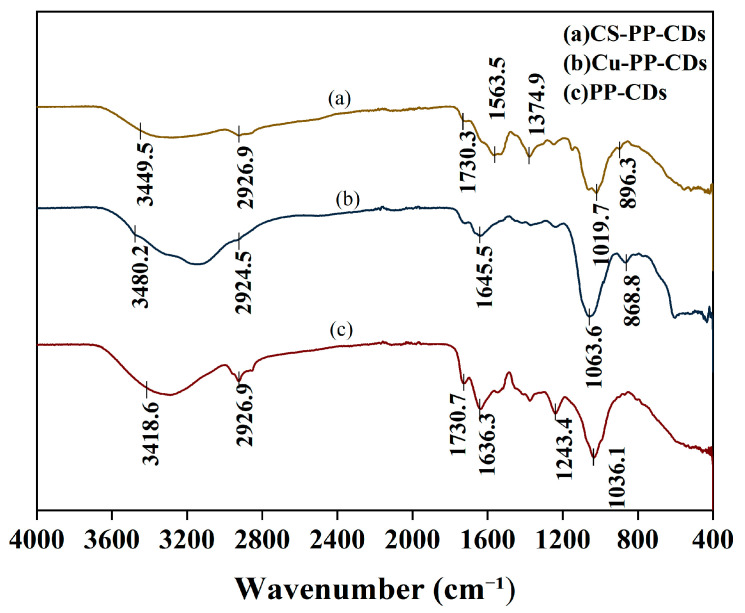
FTIR Spectra of PP-CDs, Cu-PP-CDs and CS-PP-CDs.

Shown in Figure 6 are the Fourier transform infrared spectra of three different CDs.

Surface functional groups are crucial for CDs, as they largely determine their physicochemical properties and biological effects [33]. The surface functional groups of the three CDs were characterized by Fourier transform infrared spectroscopy (FTIR) in the scanning range of 400–4000 cm^−1^, as shown in the figure above.

PP-CDs are represented by (c), (b) represents Cu-PP-CDs and (a) CS-PP-CDs. All three types exhibit broad and intense absorption peaks at 3400–3500 cm^−1^, primarily attributed to the stretching vibrations of O-H and N-H groups, indicating abundant hydrophilic functional groups such as hydroxyl and amino on the carbon dot surfaces. In the 2800–3000 cm^−1^ range, weak C-H bond stretching vibrations are observed across all three types, suggesting the presence of minor alkyl chain structures on the carbon dot surfaces. The absorption peaks in the 1600–1700 cm^−1^ range correspond to C=O bond stretching vibrations, mainly originating from oxygen-containing functional groups like carboxyl and carbonyl groups on the carbon dot surfaces. The absorption intensity of Cu-PP-CDs in this range is slightly lower than that of PP-CDs, likely due to coordination between copper ions and carboxyl groups, which reduces the number of free C=O bonds. The C=O bond absorption peak in CS-PP-CDs shows slight splitting due to the introduction of amide bonds (-CONH-) in chitosan, further confirming successful grafting of CS-PP-CD surfaces. The absorption peaks in the 1000–1300 cm^−1^ range correspond to C-O bond stretching vibrations, with all three types demonstrating significant absorption in this region.

#### 3.3.5. Comparative Study of Carbon Dot Elemental Composition

**Figure 7:**
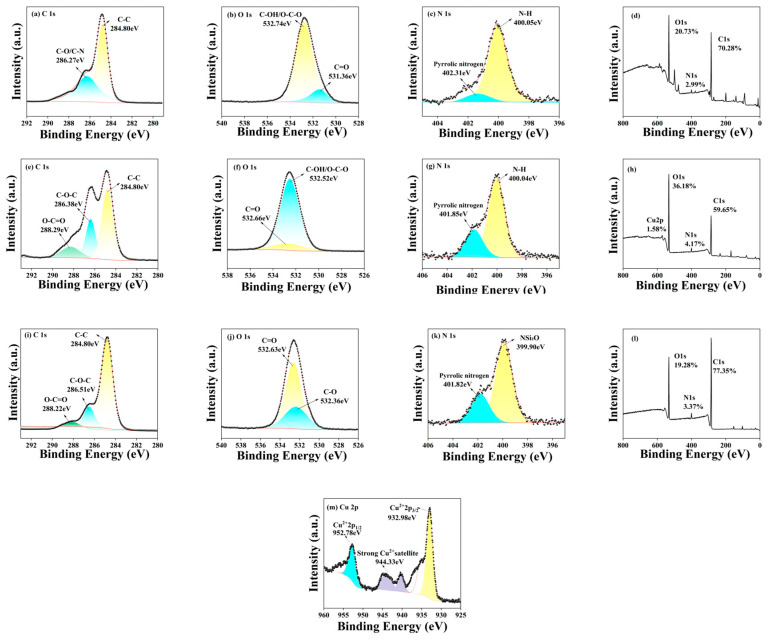
XPS Spectra of PP-CDs, Cu-PP-CDs and CS-PP-CDs. (**a**) High-resolution C 1s spectrum of PP-CDs; (**b**) High-resolution O 1s spectrum of PP-CDs; (**c**) High-resolution N 1s spectrum of PP-CDs; (**d**) XPS full-spectrum of PP-CDs; (**e**) High-resolution C 1s spectrum of Cu-PP-CDs; (**f**) High-resolution O 1s spectroscopy of Cu-PP-CDs; (**g**) High-resolution N 1s spectrum of Cu-PP-CDs; (**h**) XPS full-spectrum of Cu-PP-CDs; (**i**) High-resolution C 1s spectrum of CS-PP-CDs; (**j**) High-resolution O 1s spectroscopy of CS-PP-CDs; (**k**) High-resolution N 1s spectrum of CS-PP-CDs; (**l**) XPS full-spectrum of CS-PP-CDs; (**m**) High-resolution Cu 2p spectrum of Cu-PP-CDs.

Shown in Figure 7 are the X-ray photoelectron spectroscopy spectra of three different CDs.

While Fourier transform infrared (FTIR) spectroscopy can only reveal vibration modes of surface functional groups in CDs, X-ray photoelectron spectroscopy (XPS) analysis, when combined with FTIR data, provides additional insights into the surface elemental composition and chemical states of the three carbon dot types.

The atomic percentages of carbon (C), oxygen (O) and nitrogen (N) in PP-CDs are 70.28%, 26.73% and 2.99%, respectively; for Cu-PP-CDs, the atomic percentages of C, O and N are 59.65%, 36.18% and 4.17%, with a trace amount of copper (Cu, 1.58%); while the atomic percentages of C, O and N in CS-PP-CDs are 65.32%, 29.45% and 5.23%, respectively.

This study first employed an XPS analyzer to determine the elemental composition of PP-CDs. The C 1s spectrum (Figure 7a) reveals only C-C (284.80 eV) and C-O/C-N (286.27 eV) bonds, indicating that the CDs primarily exist as carbon-carbon skeletons bonded with oxygen or nitrogen-containing groups. The O 1s spectrum (Figure 7b) shows splitting into C-OH/C-O (532.74 eV) and C=O (531.36 eV), demonstrating the presence of oxygen-containing functional groups such as hydroxyl and carboxyl on the surface. The N 1s spectrum (Figure 7c) mainly contains N-H bonds (400.05 eV) and pyridine nitrogen (402.31 eV), indicating nitrogen exists in amino and pyridine forms. The full spectrum (Figure 7d) contains only C, O and N elements, consistent with the compositional characteristics of pure CDs [34].

Secondly, the analysis of Cu-PP-CDs revealed distinct spectral characteristics. The C 1s spectrum (Figure 7e) exhibited an additional O-C=O peak at 288.29 eV, indicating the presence of functional groups such as carboxyl groups. The O 1s spectrum (Figure 7f) showed a weakened C=O peak (532.66 eV) compared to pure CDs. Combined with the characteristic Cu 2p peak in the full spectrum (Figure 7h), this suggests that copper ions coordinate with carboxyl groups, reducing their proportion. Figure 7m presents the high-resolution XPS spectrum of Cu 2p for Cu-PP-CDs, where characteristic peaks appear at 932.98 eV and 952.76 eV, corresponding to the 2p_3_/_2_ and 2p_1_/_2_ orbitals of Cu^2+^, respectively. Meanwhile, a satellite peak of Cu^2+^ is detected at 944.36 eV, confirming that copper exists in the chemical state of divalent copper ions in the CDs; furthermore, the peak shape and binding energy data of the Cu^2+^ characteristic peaks further illustrate that copper ions are not doped in the form of simple substances but form stable coordination structures with oxygen-containing functional groups (e.g., carboxyl groups) on the surface of CDs through coordination bonds. The N 1s spectrum (Figure 7g) demonstrated no significant shift in the N-H peak, indicating that the amino groups remain uncoordinated with copper and retain their hydrophilic properties.

Furthermore, the analysis of CS-PP-CDs revealed distinct spectral characteristics. The C 1s spectrum (Figure 7i) exhibited numerous hydroxyl groups through the presence of C-O peaks. The O 1s spectrum (Figure 7j) showed elevated C=O peak intensities, suggesting the formation of amide bonds that confirm successful chitosan grafting onto the carbon dot surface. The N 1s spectrum (Figure 7k) with binding energy up to 399.90 eV indicated covalent grafting reactions between chitosan and functional groups on the carbon dot surface, resulting in altered chemical environments of nitrogen atoms. The comprehensive spectrum (Figure 7l) showed no new elements, confirming that chitosan was covalently or hydrogen-bonded to the CDs without introducing additional impurities.

The XPS analysis results corroborate the FITR findings, demonstrating the presence of functional groups such as hydroxyl, carbonyl and amino groups on the surfaces of all three types of CDs [35]. However, there are differences in content and chemical environment: the proportion of carbon element in pure CDs is higher than that in Cu-PP-CDs, but lower than that in CS-PP-CDs. The high oxygen-containing functional group modification accompanied by low carbon content gives the three good water dispersion; Cu-PP-CDs exhibit enhanced surface functional diversity through the coordination of copper ions, and the presence of divalent copper ions not only introduces new active sites to the CDs but also regulates the photophysical and chemical properties of the CDs through electron transfer, which is also an important reason for the enhancement of their catalytic and antibacterial activities [36]; CS-PP-CDs demonstrate improved structural stability via carbon skeleton reinforcement. These distinct characteristics directly reflect the doping mechanisms: pyridine nitrogen in pure CDs contributes to structural stability, copper coordination sites in Cu-PP-CDs enhance catalytic activity and abundant hydroxyl groups in CS-PP-CDs improve biocompatibility. These findings provide theoretical foundations for subsequent performance optimization and application selection. Furthermore, variations in elemental composition may induce different interfacial behaviors and biological effects [37].

The antioxidant activity of CDs mainly depends on the hydrogen atom donating capacity and electron transfer efficiency of surface oxygen-containing functional groups, both of which are regulated by elemental composition and chemical states [38]. The optimal C/O ratio of PP-CDs endows them with two core antioxidant pathways: abundant -OH and -COOH groups can directly donate hydrogen atoms to DPPH· and ABTS^+^·radicals, converting them into stable products; the conjugated carbon skeleton and pyrrolic nitrogen enhance electron mobility, which also explains why their free radical scavenging efficiency is comparable to that of vitamin C. The decreased antioxidant activity of Cu-PP-CDs stems from two aspects: the coordination between Cu^2+^ and -COOH reduces free active sites, and the empty d-orbitals of Cu^2+^ capture excited electrons in the conduction band of CDs, forming non-radiative transition channels that compete with the electron transfer process of free radicals. CS-PP-CDs exhibit moderate antioxidant activity: the hydroxyl groups of chitosan provide additional hydrogen atom donating sites, but the long-chain structure shields some active sites, and the formation of amide bonds reduces the conjugation degree of the carbon skeleton, leading to decreased electron mobility.

In summary, elemental composition and chemical states directly determine the antioxidant performance of PP-CDs: PP-CDs exhibit the optimal antioxidant activity due to their stable carbon skeleton, abundant free oxygen-containing functional groups, and enhanced electron transfer capacity; Cu-PP-CDs sacrifice part of the antioxidant performance through copper ion coordination in exchange for improved antibacterial activity; CS-PP-CDs achieve a balance between antioxidant activity and biocompatibility via chitosan modification. These findings provide support for the “functional customization” of biomass CDs, enabling their targeted design for specific application scenarios.

Pure CDs can be used as basic fluorescent materials [39]; Cu-PP-CDs, through the incorporation of Cu, can be utilized in catalytic reactions or heavy metal detection. CS-PP-CDs, owing to the biocompatibility of chitosan, are particularly suitable for biomedical applications such as bioimaging and drug delivery. Different doping methods enable CDs to adapt their functionalities to diverse application scenarios.

## 4. Discussion

Structural analysis showed that copper ions and chitosan could alter the physicochemical properties of PP-CDs, thus affecting their functionality. PP-CDs had an amorphous structure with short-range graphite-like domains, corresponding to the broad XRD peak at 2θ = 19.8° and blurred HRTEM lattice fringes. By contrast, Cu-PP-CDs formed a “crystalline-amorphous” composite structure, evidenced by sharp XRD peaks of copper-based phases and distinct HRTEM lattices, consistent with relevant studies that metal ion doping may improve carbon dot skeleton regularity and potentially generate antibacterial active sites. CS-PP-CDs exhibited a more dispersed amorphous structure due to chitosan polysaccharide chains, which might enhance dispersion uniformity and biocompatibility but reduce lattice order.

Surface hydroxyl, carboxyl and amino groups detected by FTIR and XPS correlated closely with PP-CDs’ antioxidant activity. PP-CDs were rich in oxygen-containing groups that acted as electron donors to scavenge free radicals, giving them DPPH/ABTS scavenging capacities comparable to VC. Copper ion doping caused free carboxyl groups to coordinate with Cu^2+^, possibly weakening electron transfer and reducing antioxidant activity. Chitosan doping introduced additional hydroxyl groups but its macromolecular chains might mask partial active sites, leading to moderate antioxidant performance. These results suggested that the antioxidant capacity of biomass carbon dots may relate to the types and contents of surface functional groups.

Antibacterial tests revealed a clear performance hierarchy: Cu-PP-CDs > CS-PP-CDs > pure PP-CDs. Cu-PP-CDs possessed strong antibacterial activity, with the minimum inhibitory concentration against *E. coli* at 2.5 mg/mL, potentially from synergistic effects. Given that this study did not directly quantify the release/leaching of Cu^2+^, the contribution of free Cu^2+^ to the antibacterial activity cannot be completely excluded. Therefore, copper ion release may potentially disrupt bacterial membranes; crystalline copper phases might serve as active antibacterial sites, and the carbon dot scaffold could also be involved in reactive oxygen species generation. Compared with pure PP-CDs, CS-PP-CDs exhibited enhanced antibacterial activity, which may be associated with chitosan promoting the adhesion of the material to bacterial surfaces and disrupting cell wall integrity.

Fluorescence performance analysis revealed that pristine CDs displayed the highest fluorescence emission intensity (8172.4 a.u.), while Cu-PP-CDs showed a significant reduction in fluorescence intensity (2279.4 a.u.), and CS-PP-CDs exhibited moderate fluorescence attenuation (5416.7 a.u.). This difference mainly stems from the interactions between the dopants and CDs: copper ions form coordination structures with surface functional groups of CDs, and chitosan is grafted onto the carbon dot surface via chemical bonds. Both modifications alter the fluorescence emission behavior of CDs, with copper ions exerting a more pronounced effect.

To further enhance the green credentials of the process in future research, hydrodynamic cavitation could be employed for PP-CDs extraction. Leveraging the intense localized energy released by bubble collapse, this technology would enable efficient disruption of pineapple peel cell walls without the need for high-temperature input. Compared with microwave extraction, it would offer superior energy efficiency, reduce water consumption and downstream processing requirements, while safeguarding high product yield and purity, thereby facilitating the green industrialization of PP-CDs [40].

From a prospective life cycle assessment (LCA) and techno-economic analysis (TEA) perspective, this PP-CDs synthesis route is expected to exhibit prominent environmental and economic advantages. In terms of LCA, the utilization of agricultural waste pineapple peel as raw material would realize waste valorization and reduce the environmental burden associated with agricultural waste disposal; microwave-assisted synthesis combined with optional industrial spray drying would significantly cut down total energy and water consumption, further optimizing the environmental performance of the entire life cycle. From the TEA aspect, the zero-cost raw material would lower the initial production cost, while scalable optimization technologies (e.g., spray drying, hydrodynamic cavitation extraction) would effectively reduce the unit product cost by improving production efficiency. Therefore, this route holds great potential for balancing environmental sustainability and economic viability in future large-scale production.

This study has several limitations, which will be addressed in subsequent research:

(1) In vitro evaluations cannot fully reflect performance in real-world application scenarios; future work will establish practical application systems such as food preservation and biomedicine to verify the performance of CDs in complex matrices.
(2) The long-term biotoxicity of Cu-PP-CDs requires in-depth assessment; animal models and long-term cell culture experiments are planned to systematically investigate their metabolic pathways and toxicological impacts in vitro and in vivo.
(3) The molecular mechanism underlying strain-specific antibacterial activity remains unclear; advanced techniques including transcriptomics, proteomics, and atomic force microscopy characterization will be employed to elucidate the interaction modes between CDs and bacterial cell walls/membranes.
(4) Newly added: The fluorescence stability of CDs has not been verified in complex environments; subsequent studies will test the fluorescence retention capacity of different CDs under simulated practical application conditions (e.g., light exposure, temperature variations and pH fluctuations) to provide data support for their application in fluorescence detection-related scenarios.

In summary, this work demonstrates that pineapple peel can be converted into high-performance CDs via a green microwave method. Doping modifications enable the customized regulation of carbon dot properties: pristine CDs excel in antioxidant and fluorescence performance, Cu-PP-CDs are ideal for antibacterial applications and CS-PP-CDs offer balanced multifunctionality. These findings provide a new pathway for the sustainable utilization of agricultural waste and an important strategy for the targeted design and application of biomass CDs.

## 5. Conclusions

This study utilizes pineapple peel, an agricultural by-product, as a carbon source. Through a green and efficient microwave method, we successfully synthesized pure CDs and modified CDs doped with copper ions and chitosan. The research systematically elucidated how doping modifications regulate the structural characteristics, antioxidant properties and antibacterial performance of CDs. Results demonstrate that doping optimization can specifically enhance CD functionalities. Among these, CDs exhibit optimal antibacterial activity, while CS-PP-CDs demonstrate balanced antibacterial and antioxidant potential. These findings provide theoretical foundations for the high-value utilization of pineapple peel and the customized preparation of multifunctional CDs.

(1) All three types of CDs exhibit spherical morphology with excellent dispersion. Pure CDs are predominantly amorphous, while copper doping creates a ‘crystalline-amorphous’ structure. Chitosan doping reduces lattice order. All three types contain hydroxyl and carboxyl groups, with successful integration of the doped components. The theoretical basis and technical support are provided.
(2) The antioxidant performance shows concentration-dependent characteristics. Pure PP-CDs exhibited the highest scavenging efficiency for DPPH and ABTS free radicals, achieving 50% clearance at approximately 0.6 mg/mL and 0.2 mg/mL concentrations, respectively. CS-PP-CDs followed, while Cu-PP-CDs demonstrated the weakest scavenging effect. This indicates that doping modification has a certain weakening effect on the antioxidant performance of CDs.
(3) The antibacterial performance was significantly enhanced through doping modification. Cu-PP-CD samples demonstrated superior antibacterial efficacy compared to chitosan-doped samples, both outperforming pure PP-CDs. Both doped carbon dot types exhibited stronger inhibitory effects against Gram-negative *Escherichia coli* than Gram-positive *Staphylococcus aureus*, demonstrating distinct strain-specific targeting capabilities.

This study may provide an important foundational reference and theoretical support for the functional customization of CDs. It is expected that such materials can be further designed, performance-regulated, and structurally optimized in a more targeted manner in accordance with the practical requirements and application standards of different scenarios.

Notably, although CDs and their doped derivatives show promisingly low cytotoxicity as documented in the existing literature, dedicated migration testing and comprehensive safety assessment remain indispensable prerequisites for their deployment in food-contact systems [41].

Endowed with the combined advantages of low toxicity, high safety, high antibacterial efficiency, environmental friendliness and low cost, CDs and their composite materials hold considerable application potential in the highly practical field of functional coatings for food packaging. They provide brand new ideas for the subsequent research, development and innovation of related functional protective coatings, boasting broad prospects for further in-depth exploration and practical application.

## Figures and Tables

**Table 1:** Table of comparison of antibacterial zone diameters between pineapple peel-based carbon dots and their control groups.

Strain	PP-CDs	Cu-PP-CDs	CS-PP-CDs	Chitosan	Cefotaxime	Deionized Water
*E. coli* /mm	0	23.1 ± 0.13	12.8 ± 0.08	10.2 ± 0.21	34.2 ± 0.17	0
*S. aureus* /mm	0	17.3 ± 0.05	16.3 ± 0.12	8.7 ± 0.22	35.1 ± 0.06	0

## Data Availability

The original contributions presented in this study are included in the article. Further inquiries can be directed to the corresponding author.

## Abbreviations

The following abbreviations are used in this manuscript:

CDs	Carbon Dots
PP-CDs	Pineapple Peel Carbon Dots
Cu-PP-CDs	Copper-Doped Pineapple Peel Carbon Dots
CS-PP-CDs	Chitosan-Doped Pineapple Peel Carbon Dots
DPPH	2,2-Diphenyl-1-Picrylhydrazyl
ABTS	2,2′-Azinobis-(3-Ethylbenzthiazoline-6-Sulfonic Acid)
MIC	Minimum Inhibitory Concentration
TEM	Transmission Electron Microscopy
XRD	X-Ray Diffraction
FTIR	Fourier Transform Infrared Spectroscopy
XPS	X-Ray Photoelectron Spectroscopy
VC	Vitamin C
PBS	Phosphate-Buffered Saline
